# Pathways between caregiver body mass index, the home environment, child nutritional status, and development in children with severe acute malnutrition in Malawi

**DOI:** 10.1371/journal.pone.0255967

**Published:** 2021-08-23

**Authors:** Allison I. Daniel, Mike Bwanali, Eric O. Ohuma, Celine Bourdon, Melissa Gladstone, Isabel Potani, Emmie Mbale, Wieger Voskuijl, Meta van den Heuvel, Robert H. J. Bandsma

**Affiliations:** 1 Centre for Global Child Health, Hospital for Sick Children, Toronto, Ontario, Canada; 2 Translational Medicine Program, Hospital for Sick Children, Toronto, Ontario, Canada; 3 Department of Nutritional Sciences, Temerty Faculty of Medicine, University of Toronto, Toronto, Ontario, Canada; 4 The Childhood Acute Illness & Nutrition (CHAIN) Network, Blantyre, Malawi; 5 London School of Hygiene and Tropical Medicine, London, United Kingdom; 6 Department of Women’s and Children’s Health, Institute of Translational Medicine, University of Liverpool, Liverpool, United Kingdom; 7 Department of Paediatrics, College of Medicine, University of Malawi, Blantyre, Malawi; 8 Global Child Health Group, Emma Children’s Hospital, Amsterdam University Medical Centre, University of Amsterdam, Amsterdam, The Netherlands; 9 Division of Paediatric Medicine, Hospital for Sick Children, Toronto, Ontario, Canada; 10 Department of Paediatrics, Faculty of Medicine, University of Toronto, Toronto, Ontario Canada; 11 Department of Biomedical Sciences, College of Medicine, University of Malawi, Blantyre, Malawi; Prince Sattam Bin Abdulaziz University, College of Applied Medical Sciences, SAUDI ARABIA

## Abstract

Children with severe acute malnutrition (SAM) remain vulnerable after treatment at nutritional rehabilitation units (NRUs). The objective was to assess the concurrent pathways in a hypothesized model between caregiver body mass index (BMI), the home environment, and child nutritional status, and development (gross motor, fine motor, language, and social domains) in children with SAM following discharge from inpatient treatment. Structural equation modelling (SEM) was performed with data from a cluster-randomized controlled trial at the Moyo Nutritional Rehabilitation and Research Unit in Blantyre, Malawi. This approach was undertaken to explore simultaneous relationships between caregiver BMI, the home environment (Home Observation for Measurement of the Environment Inventory scores), child nutritional status (anthropometric indicators including weight-for-age z-scores [WAZ]), and child development (Malawi Developmental Assessment Tool (MDAT) z-scores as a latent variable) in children with SAM. These data were collected at participants’ homes six months after discharge from NRU treatment. This analysis included 85 children aged 6–59 months with SAM and their caregivers recruited to the trial at the NRU and followed up successfully six months after discharge. The model with WAZ as the nutritional indicator fit the data according to model fit indices (χ^2^ = 28.92, p = 0.42). Caregiver BMI was predictive of better home environment scores (β = 0.23, p = 0.03) and child WAZ (β = 0.30, p = 0.005). The home environment scores were positively correlated with MDAT z-scores (β = 0.32, p = 0.001). Child nutritional status based on WAZ was also correlated with MDAT z-scores (β = 0.37, p<0.001). This study demonstrates that caregiver BMI could ultimately relate to child development in children with SAM, through its links to the home environment and child nutritional status.

## Introduction

In low- and middle-income countries, an estimated 250 million children are unlikely to achieve optimal cognitive, motor, and socioemotional development [1]. Children are at high risk of compromised development if they live in poverty, are exposed to an unstimulating environment, and experience acute illness or malnutrition [2–4].

Children with complicated severe acute malnutrition (SAM) are often subjected to many of these risk factors. When children have SAM in combination with an acute illness, loss of appetite, or severe oedema, they require inpatient treatment at nutritional rehabilitation units (NRUs) according to the Community Based Management of Acute Malnutrition guidelines [5]. Aside from an increased risk of mortality within two years after discharge from inpatient treatment, these children have poor nutritional outcomes including low height-for-age z-score (HAZ), representative of stunting, and inhibited gross motor, fine motor, language, and social development [6–9].

There is strong evidence supporting the association between HAZ and child development [1, 2, 10–12]. A systematic review examining the effects of nutritional supplementation and caregiving interventions have shown that improvements in HAZ are associated with better developmental outcomes, while caregiving interventions lead to better developmental outcomes but not higher HAZ [13]. The pathways between nutritional status and developmental outcomes require further exploration particularly in children with SAM. There is a particular need to understand how different anthropometric indicators like WHZ and weight-for-age z-scores (WAZ) in children with SAM predict child development.

For children to reach their developmental potential, care practices that promote health, nutrition, and early learning of children are essential. Therefore, caregivers are central in this narrative [14–17]. Based on qualitative research conducted in Malawi, Gladstone *et al* (2018) identified that caregiver well-being and care practices are important to achieve better child health and development [18]. Another qualitative study done in Malawi examined perceptions and experiences of caregivers of children admitted for inpatient treatment of SAM [19]. This study revealed a number of themes reported by caregivers that relate to difficulties in caring for their children at home, including caregiver well-being [19]. Yet specific caregiver factors such as nutritional status, which could relate to well-being, has not been a strong focus of research in caregivers of children with SAM. We hypothesized that maternal nutritional status according to body mass index (BMI) could be associated with factors such as income and the number of children in the household which could influence care practices.

Because there are many social and nutritional factors that influence developmental outcomes in children with SAM, analytic techniques that consider simultaneous pathways are advantageous. Structural equation modelling (SEM) is a type of analysis that confirms and measures structural pathways between measured and latent variables. The objective of this analysis was to evaluate concurrent pathways between caregiver body mass index (BMI), the home environment, child nutritional status according to multiple different anthropometric indicators, and development in children with SAM in the home setting following discharge from inpatient treatment. An additional objective was to understand which anthropometric indicator is most predictive of child development in terms of gross motor, fine motor, language, and social domains within this SEM. This will allow for a deeper understanding of mechanistic pathways and potential areas to intervene with the goal of having the most influence on developmental outcomes in children with SAM.

## Materials and methods

This analysis was conducted with data from a cluster-randomized controlled trial evaluating the effectiveness of a hospital-based interactive counselling intervention, known as the Kusamala Program, to improve gross motor, fine motor, language, and social development in children admitted for inpatient treatment of SAM [20, 21]. The trial was conducted at the Moyo NRU at Queen Elizabeth Central Hospital in Blantyre, Malawi. Ethical approval for this study was obtained from the University of Malawi College of Medicine Research Ethics Committee (P.04/16/1930) and the Hospital for Sick Children Research Ethics Board (1000053578). Written or verbal informed consent was obtained from all subjects/patients. Verbal consent was witnessed and formally recorded. The protocol for this trial was published and can be accessed for more details [20].

### Trial inclusion criteria

1. Child 6–59 months of age with SAM, identified by bilateral pitting oedema, WHZ below -3 SD, and/or MUAC below 115 mm;
2. Child admitted to hospital because of SAM with medical complications or appetite-loss per World Health Organization (WHO) guidelines [22];
3. Primary caregiver (self-identified) present at hospital.

### Trial exclusion criteria

1. Primary caregiver declined to give informed consent;
2. Child with a known terminal illness;
3. Child requiring a surgical procedure.

An additional exclusion criterion was applied in that children with identified neurodisability were omitted from the analysis per the original trial protocol because of the influence on developmental scores [20]. Furthermore, caregivers and children who were lost to follow-up were not included in the main analysis, but their baseline characteristics were examined in comparison to caregivers and children with follow-up data.

Screening and informed consent procedures, including written or verbal consent in the presence of a witness in the case that caregivers were unable to read or write, were completed by trained research staff members. Children and their caregivers were recruited and enrolled to the trial within three days of admission. Groups of one to six children and their caregivers were randomized each week to an intervention arm in which caregivers participated in the Kusamala Program or to a comparison arm with no intervention beyond the current practices in the NRU including basic nutrition and WASH counselling at the time of discharge [20].

### Data collection and variables for hypothesized pathway model

Data for the cluster-randomized controlled trial were collected at the time of enrollment, discharge from hospital, and at follow-up six months after discharge at the homes of caregivers and children. Baseline data included household and caregiver characteristics as well as child characteristics and anthropometric indices calculated per the 2006 WHO Child Growth Standards [23]. Caregiver and child weight and height were measured in duplicate, and if there were discrepancies in these two measurements then a third measurement was taken.

**Fig 1:**
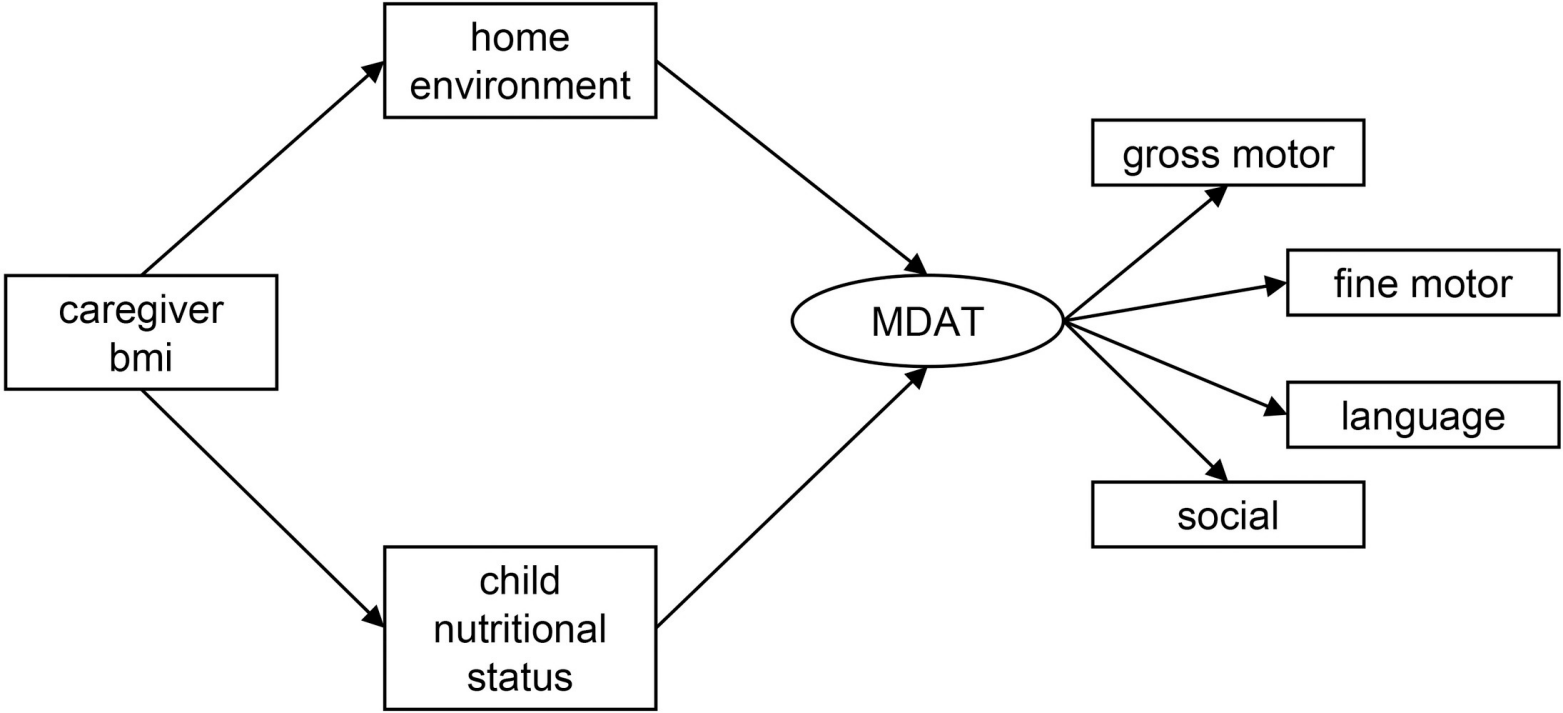
Hypothesized pathways between caregiver BMI, the home environment, child nutritional status and development in children with SAM. Caregiver body mass index (bmi); home environment according to the Home Observation for Measurement of the Environment Inventory; child nutritional status according to weight-for-height z-scores (whz), weight-for-age z-scores (waz), height-for-age z-scores (haz), or mid-upper arm circumference (muac); and development according to MDAT (Malawi Developmental Assessment Tool) z-scores.

An *a priori* hypothesized pathway model was developed with specific pathways leading to child development with all data in the model from follow-up at six months after discharge (Fig 1). Specific variables in the model were pre-selected as they were hypothesized to relate to developmental outcomes directly or indirectly in children with SAM. Caregiver BMI is the exogenous variable in the model leading to the home environment and to child nutritional status. This analysis evaluated four different anthropometric indicators of nutritional status in different versions of the SEM. WHZ and MUAC are both indicators of wasting [23]. WAZ, an indicator of underweight status, was also examined [23]. HAZ was computed to indicate linear growth relative to the child’s age [23].

The Home Observation for Measurement of the Environment (HOME) Inventory, which has been widely used in low- and middle-income settings, was adapted for relevance to this particular study population and included 18 observations of care practices and seven interview questions answered by caregivers [24, 25]. The HOME Inventory used included 25 items evaluating responsivity (11 items), language stimulation (2 items), acceptance (6 items), learning materials (1 item), involvement (3 items), and variety (2 items) [24, 25].

The home environment and child nutritional status are both hypothesized to be directly linked to child development as the main outcome. Child development is represented by a combination of developmental domains as a latent variable including gross motor, fine motor, language, and social development z-scores according to the Malawi Developmental Assessment Tool (MDAT) [26]. The MDAT is a culturally validated measure of child development specifically for use in low-resource African settings and has been used in several recent studies [26–29]. It included 36 items in each of four domains including gross motor, fine motor, language, and social development. The MDAT has good reliability for all domains and is sensitive to nutritional status as indicated by 28% of malnourished children passing the test using a pass/fail technique compared to 94% of non-malnourished passing in a previous validation study [26]. MDAT z-scores for each respective domain were calculated by comparing scores to a reference population in Malawi [26].

### Statistical analysis

All data were entered into a Research Electronic Data Capture database and subsequently analyzed in Stata 16 (Statacorp LP, College Station, Texas, USA) [30, 31]. Descriptive statistics were computed including means (SD) or median (interquartile range) for continuous variables as appropriate, and proportions and percentages for categorical variables. The *zscore06* macro was used in Stata 16 to calculate anthropometric z-scores per the 2006 WHO Child Growth Standards [23, 32]. MDAT z-scores were computed based on a reference population of Malawian children [26, 33]. HAZ and MDAT z-scores were considered valid if they were within -6 and +6 SD, though for WHZ and WAZ they were considered between -6 SD and +5 SD [23].

Characteristics of children who died or were lost to follow-up were compared to surviving children who were successfully followed up using one-sample t-tests for continuous variables and Pearson’s χ^2^ test for categorical variables. P-values below 0.05 were considered statistically significant for all analyses. Children who died during inpatient treatment or within six months after discharge were excluded from the main analysis for this paper which examined follow-up data only.

Multiple linear regression was done to characterize and understand factors related to caregiver BMI as the exogenous variable in the hypothesized pathway model. Predictor variables included monthly household income, caregiver age, caregiver height, and number of children in the household. Standardized beta-coefficients were examined for this analysis.

This analysis included all participants followed up in the first three years of the cluster-randomized controlled trial, between November 2016 and November 2019, to reach a target sample size exceeding 70 children and caregivers with follow-up data for ten observations per variable in the SEM [34–36]. Q–Q plots were examined for each variable in the SEM to check that the residuals were normally distributed (S1 Fig).

SEM was then done to evaluate the hypothesized pathway model, which first involved confirmatory factor analysis (CFA) of MDAT as a latent variable. The full information maximum likelihood method was used for the SEM to account for missing data. Covariates in the model included child human immunodeficiency virus (HIV) status, sex, and age. Standardized beta-coefficients were computed for all pathways and their direct and indirect effects were calculated. Furthermore, simple linear regression was done to assess the relationships between individual variables in the SEM.

Model fit for the SEM was examined using the χ^2^ test statistic (χ^2^ p-value >0.05 acceptable, indicating no statistically significant difference between the hypothesized and fitted model), comparative fit index (CFI >0.90 represents good fit), Tucker-Lewis index (TLI >0.90 represents good fit), and root mean square error for approximation (RMSEA <0.05 represents good fit, <0.08 acceptable fit) [37–39]. Bootstrap estimates and confidence intervals were also evaluated by completing SEM on the estimation sample to understand the stability of the model and to further verify that the sample size was adequate.

### Reporting guidelines

The Strengthening the Reporting of Observational Studies in Epidemiology statement was followed in the reporting of this manuscript [40].

## Results

After screening for eligibility, 170 children and their caregivers were enrolled in the first three years of the cluster-randomized controlled trial (S2 Fig). Eight participants were excluded because they were deemed ineligible for the trial after enrollment (3/170, 1.8%) or withdrew from the study (5/170, 3.0%). Twenty-seven children (27/162, 16.7%) were excluded from the analysis due to pre-existing neurodisability. Of the remaining children, there were 18 inpatient deaths (18/135, 13.3%). In children that survived inpatient treatment, there were 17 losses to follow-up (17/117, 14.5%) in addition to 15 outpatient deaths (15/100, 15.0%). This left 85 children and their caregivers available for analysis. The mean follow-up time was 6.4 months after discharge. A small proportion of data were missing for certain variables (S1 Table).

### Baseline characteristics

Child, caregiver, and household characteristics at baseline are summarized in Table 1. Children who died had lower WHZ compared to those who survived and were successfully followed up (-4.0 ± 0.8 versus -3.4 ± 1.2, p = 0.046). The proportion of children who died that had HIV tended to be higher compared to surviving children with follow-up data (38.7% versus 26.5%, p = 0.2). WAZ also appeared lower in children who died compared to those that survived and were followed up (-4.6 ± 0.8 versus -3.9 ± 1.4, p = 0.10). Baseline characteristics between participants successfully followed up and those lost to follow-up were similar. However, caregivers that were lost to follow-up had a lower average number of children in the household compared to those that were followed up (2.2 ± 1.3 versus 3.0 ± 1.4, p = 0.03).

**Table 1:** Baseline characteristics of children admitted for inpatient treatment of SAM and their caregivers and households.

	Children with follow-up data (n = 85)	Children who died (n = 33)	Children lost to follow-up (n = 17)
**Age (months), mean (SD)**	19.5 (9.8)	19.8 (12.6)	22.8 (12.7)
**Sex female (%)**	41/85 (48.2)	17/33 (51.5)	8/17 (47.1)
**HIV positive (%)**	22/83 (26.5)	12/31 (38.7)	6/16 (37.5)
**Previous inpatient admission (%)**	12/84 (14.3)	5/30 (16.7)	2/16 (12.5)
**Oedema (%)**	28/85 (32.9)	10/32 (31.3)	9/16 (56.3)
**MUAC (cm)** ^**a**^ **, mean (SD)**	10.9 (1.1)	10.5 (1.0)	10.5 (0.9)
**WHZ** ^**a**^ **, mean (SD)**	-3.4 (1.2)	-4.0 (0.8)	-3.5 (1.3)
**HAZ, mean (SD)**	-3.1 (1.3)	-3.1 (1.8)	-2.6 (2.2)
**WAZ** ^**a**^ **, mean (SD)**	-3.9 (1.4)	-4.6 (0.8)	-4.3 (0.8)
	**Caregivers with follow-up data (n = 85)**	**Caregivers of children who died (n = 33)**	**Caregivers lost to follow-up (n = 17)**
**Relationship to child (%)**			
**Mother**	80/85 (94.1)	31/32 (96.9)	16/17 (94.1)
**Other**	5/85 (5.9)	1/32 (3.1)	1/17 (5.9)
**Age (years), mean (SD)**	28.0 (7.8)	25.8 (6.7)	24.7 (6.3)
**MUAC (cm), mean (SD)**	26.1 (3.5)	26.1 (2.6)	25.0 (2.3)
**Body mass index (kg/m** ^**2**^ **), mean (SD)**	22.3 (4.2)	22.3 (2.7)	21.5 (2.6)
**Caregiver education (%)**			
**Preschool**	18/85 (21.2)	2/32 (6.3)	1/17 (5.9)
**Primary**	41/85 (48.2)	20/32 (62.5)	10/17 (58.8)
**Secondary**	26/85 (30.6)	9/32 (28.1)	6/17 (35.3)
**Higher**	0/85 (0)	1/32 (3.1)	0 (0)
**Caregiver depressive symptoms (Self-Reporting Questionnaire 20), median (IQR)**	3.0 (7.0)	3.5 (7.5)	3.0 (8.5)
**Monthly household income (Malawi kwacha), mean (SD)**	20451 (24661)	16015 (11812)	24812 (35501)
**Number of children in the household, mean (SD)**	3.0 (1.4)	2.9 (1.5)	2.2 (1.3)
**Household area (%)**			
**Urban**	50/85 (58.8)	22/32 (68.8)	14/17 (82.4)
**Rural**	35/85 (41.2)	10/32 (31.3)	3/17 (17.7)

HAZ, height-for-age z-score. HIV, human immunodeficiency virus. MUAC, mid-upper arm circumference. SAM, severe acute malnutrition. SD, standard deviation. WAZ, weight-for-age z-score. WHZ, weight-for-height z-score.

^a^Excluding children with oedema.

### Caregiver body mass index

Multiple linear regression results showed no significant relationships for monthly household income (β = 0.22, p = 0.064), caregiver age (β = -0.026, p = 0.83), caregiver height (β = -0.14, p = 0.23), or the number of children in the household (β = 0.17, p = 0.16) as predictors of caregiver BMI.

### Latent variable of child development

Child development represented as a latent variable of MDAT z-scores showed acceptable model fit based on the CFA (χ^2^ = 3.21, p = 0.20, CFI = 0.994, TLI = 0.983, RMSEA = 0.085). All four MDAT domains had significant factor loadings in the measurement model (β = 0.80, p<0.001 for gross motor, β = 0.87, p<0.001 for fine motor, β = 0.84, p<0.001 for language, and β = 0.92, p<0.001 for social).

### Structural equation modelling results

**Fig 2:**
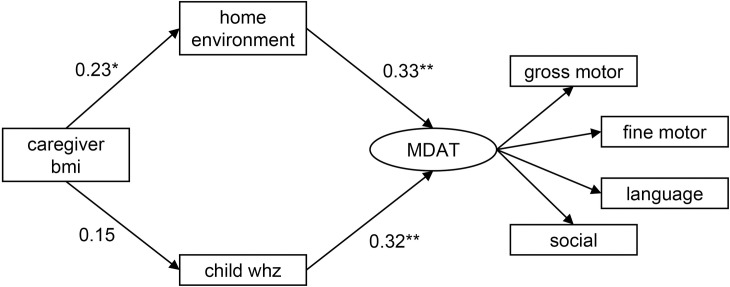
Pathways between caregiver BMI, the home environment, child nutritional status according to WHZ and development in children with SAM. Caregiver body mass index (bmi); home environment according to the Home Observation for Measurement of the Environment Inventory; child nutritional status according to weight-for-height z-scores (whz); and development according to MDAT (Malawi Developmental Assessment Tool) z-scores. Estimates represent standardized beta-coefficients. Analysis adjusted for child HIV status, sex, and age. *p<0.05, **p<0.01, ***p<0.001.

The model with WHZ as the nutritional indicator fit the data based on the various fit indices (χ^2^ = 33.99, p = 0.20, CFI = 0.976, TLI = 0.962, RMSEA = 0.050) (Fig 2 and Table 2). All pathways in the model were significant except from caregiver BMI to child WHZ (β = 0.15, p = 0.21). There was also an indirect pathway from caregiver BMI to MDAT z-scores (β = 0.12, p = 0.046), mediated by the child WHZ and home environment variables. The results of the SEM remained the same with bootstrapping, demonstrating the stability of the model.

**Table 2:** Standardized beta-coefficients and bootstrap results for direct pathways between caregiver BMI, the home environment, child nutritional status according to WHZ, and development in children with SAM.

	Structural equation modelling results			Bootstrap results		
Pathways	β	P-values	95% confidence interval	β	P-values	95% confidence interval
**home environment**						
**caregiver bmi**	0.23	0.03	0.027, 0.44	0.23	0.03	0.018, 0.46
**child whz**						
**caregiver bmi**	0.15	0.21	-0.086, 0.38	0.15	0.20	-0.078, 0.37
**MDAT**						
**home environment**	0.33	0.001	0.14, 0.52	0.33	0.001	0.14, 0.51
**child whz**	0.32	0.001	0.13, 0.51	0.32	0.003	0.11, 0.53

β, beta-coefficient (standardized). bmi, body mass index. MDAT, Malawi Developmental Assessment Tool. whz, weight-for-height z-score.

Analysis adjusted for child HIV status, sex, and age.

**Fig 3:**
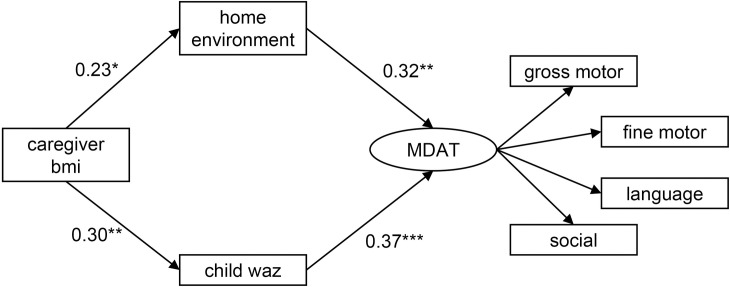
Pathways between caregiver BMI, the home environment, child nutritional status according to WAZ, and development in children with SAM. Caregiver body mass index (bmi); home environment according to the Home Observation for Measurement of the Environment Inventory; child nutritional status according to weight-for-age z-scores (waz); and development according to MDAT (Malawi Developmental Assessment Tool) z-scores. Estimates represent standardized beta-coefficients. Analysis adjusted for child HIV status, sex, and age. *p<0.05, **p<0.01, ***p<0.001.

SEM was then done to evaluate the hypothesized pathway model with WAZ as an indicator of nutritional status in place of WHZ (Fig 3 and Table 3). This model also fit the data well (χ^2^ = 31.89, p = 0.28, CFI = 0.985, TLI = 0.975, RMSEA = 0.041). In this SEM, all pathways were significant, including an indirect pathway between caregiver BMI and the MDAT latent variable (β = 0.19, p = 0.008). Results were similar upon completing bootstrapping indicating model stability.

**Table 3:** Standardized beta-coefficients and bootstrap results for direct pathways between caregiver BMI, the home environment, child nutritional status according to WAZ, and development in children with SAM.

	Structural equation modelling results			Bootstrap results		
Pathways	β	P-values	95% confidence interval	β	P-values	95% confidence interval
**home environment**						
**caregiver bmi**	0.23	0.03	0.029, 0.44	0.23	0.008	0.061, 0.46
**child waz**						
**caregiver bmi**	0.30	0.005	0.089, 0.52	0.30	0.005	0.094, 0.51
**MDAT**						
**home environment**	0.32	0.001	0.13, 0.51	0.32	0.002	0.12, 0.52
**child waz**	0.37	<0.001	0.18, 0.55	0.37	0.001	0.16, 0.58

β, beta-coefficient (standardized). bmi, body mass index. MDAT, Malawi Developmental Assessment Tool. waz, weight-for-age z-score.

Analysis adjusted for child HIV status, sex, and age.

**Fig 4:**
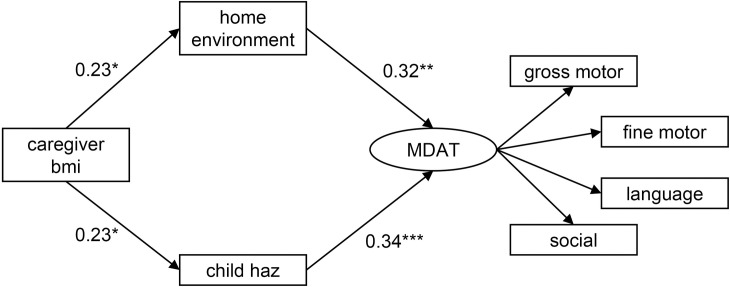
Pathways between caregiver BMI, the home environment, child nutritional status according to HAZ, and development in children with SAM. Caregiver body mass index (bmi); home environment according to the Home Observation for Measurement of the Environment Inventory; child nutritional status according to height-for-age z-scores (haz); and development according to MDAT (Malawi Developmental Assessment Tool) z-scores. Estimates represent standardized beta-coefficients. Analysis adjusted for child HIV status, sex, and age. *p<0.05, **p<0.01, ***p<0.001.

The third version of SEM was completed using HAZ as an indicator of nutritional status (Fig 4 and Table 4). This version of the model also fit the data based on the model fit indices (χ^2^ = 28.92, p = 0.42, CFI = 0.996, TLI = 0.994, RMSEA = 0.020). All pathways were significant, including the indirect pathway from caregiver BMI to the MDAT latent variable (β = 0.15, p = 0.014), and bootstrapping indicated that the model was stable.

**Table 4:** Standardized beta-coefficients and bootstrap results for direct pathways between caregiver BMI, the home environment, child nutritional status according to HAZ, and development in children with SAM.

	Structural equation modelling results			Bootstrap results		
Pathways	β	P-values	95% confidence interval	β	P-values	95% confidence interval
**home environment**	0.23	0.03	0.029, 0.44	0.23	0.01	0.050, 0.41
**caregiver bmi**						
**child haz**	0.23	0.03	0.024, 0.44	0.23	0.008	0.060, 0.41
**caregiver bmi**						
**MDAT**						
**home environment child haz**	0.32	0.001	0.14, 0.51	0.32	0.002	0.12, 0.53
	0.34	<0.001	0.16, 0.53	0.34	0.02	0.047, 0.64

β, beta-coefficient (standardized). bmi, body mass index. haz, height-for-age z-score. MDAT, Malawi Developmental Assessment Tool.

Analysis adjusted for child HIV status, sex, and age.

Lastly, MUAC was evaluated as an indicator of nutritional status, yet this SEM differed from the other three versions in that there was no direct relationship between child MUAC and the MDAT latent variable (β = 0.14, p = 0.19) (S3 Fig and S2 Table). There was a small indirect relationship between caregiver BMI and the MDAT latent variable (β = 0.10, p = 0.043).

Summary statistics of the variables in the SEM are included in S3 Table and simple linear regression with individual pathways are shown in S4 Table.

## Discussion

Using SEM, we have demonstrated that caregiver BMI, the home environment, and child nutritional status are determinants of development in children with SAM in the home setting in Malawi. There have been few studies evaluating child development in this population following discharge from NRU care, and this analysis therefore adds to the limited yet highly important body of literature around interventions beyond the inpatient period [41]. Furthermore, this analysis is innovative in that multiple pathways were explored simultaneously to identify those with the greatest influence on child development. The strongest individual pathway, based on the magnitude of the standardized beta-coefficients, in the different versions of the SEM was from child WAZ to the MDAT latent variable. However, all pathways between maternal BMI and child development were shown to be important according to the SEM results, including the indirect effect of maternal BMI on the MDAT latent variable.

These data therefore support the idea that interventions around caregiver nutritional status could be a strategy that not only addresses their own health, but also improves their ability to provide nurturing care to children with SAM. As mentioned previously, qualitative research in Malawi found that caregivers of children with SAM experience barriers to child care relating to their own well-being [19]. However, the multiple linear regression results evaluating household income, caregiver age and height, and number of children in the household did not give insight into predictors of maternal BMI. More exploration should be done to understand causes of variability and potential areas of intervention around maternal nutritional status in this population.

In this SEM, there was a direct association between the home environment and the MDAT latent variable. Previous research has also supported similar relationships between HOME Inventory scores and child development and nutritional status. For example, a caregiving and feeding intervention in Bangladesh improved child WHZ and language development, and caregivers who participated in this intervention had better HOME Inventory scores [42]. Similarly, a cluster-randomized controlled trial also done in Bangladesh evaluating a psychosocial stimulation and nutritional supplementation intervention specifically in children with SAM found that HOME Inventory scores were associated with Bayley Scales of Infant and Toddler Development cognitive scores [43]. However, our current study is the first to date that directly evaluated the HOME Inventory in relation to the MDAT, a context-appropriate indicator of child development, and examined this pathway in relation to other concurrent predictors of development [26].

Caregiver BMI was also predictive of child WAZ, HAZ, and MUAC, but not child WHZ. Of the different versions of the SEM, WAZ was the strongest anthropometric indicator predicting MDAT z- scores. Previous epidemiological data indicate that children who have both low WHZ and HAZ are at high risk of death [44, 45]. Further clinical studies in infants with SAM have also shown that low WAZ is predictive of inpatient and post-discharge mortality while WHZ is not a strong predictor [46, 47]. However, WAZ has not been widely evaluated in relation to other outcomes in children with SAM such as child development. The findings from this SEM indicate that WAZ should be considered when evaluating the consequences of nutritional deprivation on outcomes like child development and for establishing potential interventions that focus both on short- and long-term nutritional status.

We also considered exploring caregiver depressive symptoms based on the Self-Reporting Questionnaire 20 (SRQ-20), which has been translated and validated for use in Malawi [48]. However, we found that caregivers did not typically have high SRQ-20 scores, which reflect the number of depressive symptoms reported, which aligns with another study done with caregivers of children admitted to the same NRU [49]. Furthermore, past research indicated that caregiver depressive symptoms did not predict child weight gain four weeks after discharge from the NRU [50].

One of the limitations of this particular analysis is that it included children who survived inpatient treatment for SAM and lived until follow-up six months later, and the strength of the various pathways may differ for children with SAM who die during or after discharge from NRUs. The baseline differences between children who died and survived were explored and those who died were most likely to have low WHZ. Another limitation is that the sample size was relatively small. However, relationships in the SEM were significant with reasonably large beta-coefficients, model fit was good, and stability was established with bootstrapping, suggesting that the sample size was adequate to examine the hypothesized pathway model [34–36].

## Conclusions

This analysis showed the pathways and their relative importance between caregiver BMI, the home environment, and nutritional status in predicting MDAT z-scores of gross motor, fine motor, language, and social domains in children with SAM. Child WAZ was the strongest of the anthropometric indices in predicting MDAT z-scores. Interventions aimed at improving developmental trajectories of these children following discharge from inpatient treatment at NRUs should also consider addressing caregiver nutritional status. Future research should be done to evaluate the effects of social and nutritional interventions on these pathways leading to development in children with SAM.

## Supporting information

S1 Checklist (DOCX)

S1 Fig Q–Q plots for variables in the structural equation model. (PDF)

S2 Fig Study flow chart for structural equation model analysis. (PDF)

S3 Fig Pathways between caregiver BMI, the home environment, child nutritional status according to MUAC, and development in children with SAM. Caregiver body mass index (bmi); home environment according to the Home Observation for Measurement of the Environment Inventory; dietary diversity according to a 24-hour dietary recall; child nutritional status according to mid-upper arm circumference (muac); and development according to MDAT (Malawi Developmental Assessment Tool) z-scores. Estimates represent standardized beta-coefficients. Analysis adjusted for child HIV status, sex, and age. *p<0.05, **p<0.01.
(PDF)

S1 Table Missing data from participants in the structural equation model. bmi, body mass index. haz, height-for-age z-score. home, Home Observation for Measurement of the Environment. MDAT, Malawi Developmental Assessment Tool. muac, mid-upper arm circumference. waz, weight-for-age z-score. whz, weight-for-height z-score.
(PDF)

S2 Table Standardized beta-coefficients and bootstrap results for direct pathways between caregiver BMI, the home environment, child nutritional status according to MUAC, and development in children with SAM. β, beta-coefficient (standardized). bmi, body mass index. MDAT, Malawi Developmental Assessment Tool. muac, mid-upper arm circumference. Analysis adjusted for child HIV status, sex, and age.
(PDF)

S3 Table Summary statistics of the variables in the hypothesized pathway model. bmi, body mass index. haz, height-for-age z-score. home, Home Observation for Measurement of the Environment. MDAT, Malawi Developmental Assessment Tool. muac, mid-upper arm circumference. waz, weight-for-age z-score. whz, weight-for-height z-score.
(PDF)

S4 Table Linear regression results for individual pathways in the hypothesized pathway model. β, beta-coefficient (standardized). haz, height-for-age z-score. mdat, Malawi Developmental Assessment Tool. muac, mid-upper arm circumference. waz, weight-for-age z-score. whz, weight-for-height z-score.
(PDF)

## References

[1] Lu C, Black MM, Richter LM. Risk of poor development in young children in low-income and middle-income countries: an estimation and analysis at the global, regional, and country level. 2016;4(12): e916–e922. 10.1016/S2214-109X(16)30266-2 PMC5881401 27717632

[2] Grantham-McGregor SM, Cheung YB, Cueto S, Glewwe P, Richter L, Strupp B. Developmental potential in the first 5 years for children in developing countries. Lancet. 2007;369(9555): 60–70. doi: 10.1016/S0140-6736(07)60032-4 17208643 PMC2270351

[3] Aboud FE, Yousafzai AK. Global health and development in early childhood. Annu Rev Psychol. 2015;66: 433–57. doi: 10.1146/annurev-psych-010814-015128 25196276

[4] Walker SP, Wachs TD, Gardner JM, Lozoff B, Wasserman GA, Pollitt E, et al. Child development: risk factors for adverse outcomes in developing countries. Lancet. 2007;369(9556): 145–57. doi: 10.1016/S0140-6736(07)60076-2 17223478

[5] World Health Organization. Community-based management of severe acute malnutrition: A Joint Statement by the World Health Organization, the World Food Programme, the United Nations System Standing Committee on Nutrition and the United Nations Children’s Fund. 2007. Available from: https://www.who.int/nutrition/topics/Statement_community_based_man_sev_acute_mal_eng.pdf

[6] Kerac M, Bunn J, Chagaluka G, Bahwere P, Tomkins A, Collins S, et al. Follow-up of post-discharge growth and mortality after treatment for severe acute malnutrition (FuSAM study): A prospective cohort study. PLoS One. 2014;9(6): 1–10. doi: 10.1371/journal.pone.0096030 24892281 PMC4043484

[7] O’Sullivan NP, Lelijveld N, Rutishauser-Perera A, Kerac M, James P. Follow-up between 6 and 24 months after discharge from treatment for severe acute malnutrition in children aged 6–59 months: A systematic review. PLoS One. 2018;13(8): e0202053. doi: 10.1371/journal.pone.0202053 30161151 PMC6116928

[8] Van den Heuvel M, Voskuijl W, Chidzalo K, Kerac M, Reijneveld SA, Bandsma R, et al. Developmental and behavioural problems in children with Severe Acute Malnutrition in Malawi: A cross–sectional study. J Glob Health. 2017;7(2): 1–10. doi: 10.7189/jogh.07.020416 29302321 PMC5735778

[9] Schoenbuchner SM, Dolan C, Mwangome M, Hall A, Richard SA, Wells JC, et al. The relationship between wasting and stunting: a retrospective cohort analysis of longitudinal data in Gambian children from 1976 to 2016. Am J Clin Nutr. 2019;110(2): 498–507. doi: 10.1093/ajcn/nqy326 30753251 PMC6669055

[10] Walker SP, Chang SM, Wright A, Osmond C, Grantham-McGregor SM. Early childhood stunting is associated with lower developmental levels in the subsequent generation of children. J Nutr. 2015;145(4): 823–828. doi: 10.3945/jn.114.200261 25833785

[11] Sudfeld CR, McCoy DC, Fink G, Muhihi A, Bellinger DC, Masanja H, et al. Malnutrition and Its Determinants Are Associated with Suboptimal Cognitive, Communication, and Motor Development in Tanzanian Children. J Nutr. 2015;145(12): 2705–14. doi: 10.3945/jn.115.215996 26446481

[12] Grantham-McGregor SM, Powell CA, Walker SP, Himes JH. Nutritional supplementation, psychosocial stimulation, and mental development of stunted children: the Jamaican Study. Lancet. 1991;338(8758): 1–5. doi: 10.1016/0140-6736(91)90001-6 1676083

[13] Prado EL, Larson LM, Cox K, Bettencourt K, Kubes JN, Shankar AH. Do effects of early life interventions on linear growth correspond to effects on neurobehavioural development? A systematic review and meta-analysis. Lancet Glob Heal. 2019;7(10): e1398–e1413. doi: 10.1016/S2214-109X(19)30361-4 31537370

[14] Britto PR, Lye SJ, Proulx K, Yousafzai AK, Matthews SG, Vaivada T, et al. Nurturing care: promoting early childhood development. Lancet. 2017;389(10064): 91–102. doi: 10.1016/S0140-6736(16)31390-3 27717615

[15] Black MM, Aboud FE. Responsive feeding is embedded in a theoretical framework of responsive parenting. J Nutr. 2011;141(3): 490–494. doi: 10.3945/jn.110.129973 21270366 PMC3040905

[16] Engle PL, Black MM, Behrman JR, Cabral de Mello M, Gertler PJ, Kapiriri L, et al. Strategies to avoid the loss of developmental potential in more than 200 million children in the developing world. Lancet. 2007;369(9556): 229–42. doi: 10.1016/S0140-6736(07)60112-3 17240290

[17] World Health Organization, United Nations Children’s Fund, World Bank Group. Nurturing care for early childhood development: Linking survive and thrive to transform health and human potential. Geneva; 2018. Available from: http://apps.who.int/iris/bitstream/handle/10665/272603/9789241514064-eng.pdf

[18] Gladstone M, Phuka J, Mirdamadi S, Chidzalo K, Chitimbe F, Koenraads M, et al. The care, stimulation and nutrition of children from 0–2 in Malawi—Perspectives from caregivers; “Who’s holding the baby?” John-Stewart GC, editor. PLOS ONE. 2018;13(6): 489. doi: 10.1371/journal.pone.0199757 29949636 PMC6021079

[19] Gleadow Ware S, Daniel AI, Bandawe C, Mulaheya YP, Nkunika S, Nkhoma D, et al. Perceptions and experiences of caregivers of severely malnourished children receiving inpatient care in Malawi: an exploratory study. Malawi Med J. 2018;30(3): 168–174. 10.4314/mmj.v30i3.7 PMC6307046 30627351

[20] Daniel AI, van den Heuvel M, Voskuijl WP, Gladstone M, Bwanali M, Potani I, et al. The Kusamala Program for primary caregivers of children 6–59 months of age hospitalized with severe acute malnutrition in Malawi: study protocol for a cluster-randomized controlled trial. Trials. 2017;18(550): 1–11. doi: 10.1186/s13063-017-2299-3 29149905 PMC5693531

[21] Daniel AI, Bwanali M, Tenthani JC, Gladstone M, Voskuijl W, Potani I, et al. A Mixed Methods Cluster-Randomized Controlled Trial of A Hospital-Based Psychosocial Stimulation and Counseling Program For Caregivers and Children With Severe Acute Malnutrition. Curr Dev Nutr. 2021. 10.1093/cdn/nzab100 PMC8382273 34447897

[22] World Health Organization. WHO Child Growth Standards and the Identification of Severe Acute Malnutrition in Infants and Children. WHO Libr. Geneva; 2009. Available from: https://apps.who.int/iris/bitstream/handle/10665/44129/9789241598163_eng.pdf 24809116

[23] WHO Multicentre Growth Reference Study Group. WHO Child Growth Standards: Length/height-for-age, weight-for-age, weight-for-length, weight-for-height and body mass index-for-age: Methods and development. World Heal Organ Geneva. 2006. Available from: http://hpps.kbsplit.hr/hpps-2008/pdf/dok03.pdf

[24] Linver M, Brooks-Gunn J, Cabrera N. The Home Observation for Measurement of the Environment (HOME) Inventory: The Derivation of Conceptually Designed Subscales. Parenting. 2004;4(2): 99–114.

[25] Bradley RH, Caldwell BM. The HOME Inventory and family demographics. Dev Psychol. 1984;20(2): 315–320.

[26] Gladstone M, Lancaster GA, Umar E, Nyirenda M, Kayira E, van den Broek NR, et al. The Malawi Developmental Assessment Tool (MDAT): The creation, validation, and reliability of a tool to assess child development in rural African settings. PLoS Med. 2010;7(5): 1–14. 10.1371/journal.pmed.1000273 PMC2876049 20520849

[27] Ntozini R, Chandna J, Evans C, Chasekwa B, Majo FD, Kandawasvika G, et al. Early child development in children who are HIV-exposed uninfected compared to children who are HIV-unexposed: observational sub-study of a cluster-randomized trial in rural Zimbabwe. J Int AIDS Soc. 2020;23(5): 1–10. doi: 10.1002/jia2.25456 32386127 PMC7318086

[28] Prado EL, Maleta K, Caswell BL, George M, Oakes LM, DeBolt MC, et al. Early Child Development Outcomes of a Randomized Trial Providing 1 Egg Per Day to Children Age 6 to 15 Months in Malawi. J Nutr. 2020;150(7): 1933–1942. doi: 10.1093/jn/nxaa088 32286620 PMC7330477

[29] Namazzi G, Hildenwall H, Mubiri P, Hanson C, Nalwadda C, Nampijja M, et al. Prevalence and associated factors of neurodevelopmental disability among infants in eastern Uganda: A population based study. BMC Pediatr. 2019;19(1): 1–10. doi: 10.1186/s12887-018-1376-4 31651279 PMC6813088

[30] StataCorp. Stata Statistical Software: Release 16. College Station, TX: StataCorp LLC; 2019.

[31] Harris PA, Taylor R, Thielke R, Payne J, Gonzalez N, Conde JG. Research electronic data capture (REDCap)-A metadata-driven methodology and workflow process for providing translational research informatics support. J Biomed Inform. 2009;42(2): 377–381. doi: 10.1016/j.jbi.2008.08.010 18929686 PMC2700030

[32] Leroy J. ZSCORE06: Stata module to calculate anthropometric z-scores using the 2006 WHO child growth standards. Boston College Department of Economics; 2011. Available from: https://ideas.repec.org/c/boc/bocode/s457279.html

[33] Cheung YB, Gladstone M, Maleta K, Duan X, Ashorn P. Comparison of four statistical approaches to score child development: A study of Malawian children. Trop Med Int Heal. 2008;13(8): 987–993. doi: 10.1111/j.1365-3156.2008.02104.x 18554248

[34] Bentler PM, Chou CP. Practical Issues in Structural Modeling. Sociol Methods Res. 1987;16(1): 78–117.

[35] Wolf EJ, Harrington KM, Clark SL, Miller MW. Sample Size Requirements for Structural Equation Models. Educ Psychol Meas. 2013;73(6): 913–934. doi: 10.1177/0013164413495237 25705052 PMC4334479

[36] Sideridis G, Simos P, Papanicolaou A, Fletcher J. Using Structural Equation Modeling to Assess Functional Connectivity in the Brain: Power and Sample Size Considerations. Educ Psychol Meas. 2014;74(5): 733–758. doi: 10.1177/0013164414525397 25435589 PMC4245025

[37] MacCallum RC, Browne MW, Sugawara HM. Power Analysis and Determination of Sample Size for Covariance Structure Modeling. Psychol Methods. 1996;1(2): 130–149.

[38] Hu L, Bentler PM. Cutoff criteria for fit indexes in covariance structure analysis: Conventional criteria versus new alternatives. Struct Equ Model A Multidiscip J. 1999;6(1): 1–55.

[39] Bentler PM. Comparative fit indexes in structural models. Psychol Bull. 1990;107(2): 238–46. doi: 10.1037/0033-2909.107.2.238 2320703

[40] von Elm E, Altman DG, Egger M, Pocock SJ, Gøtzsche PC, Vandenbroucke JP. The strengthening the reporting of observational studies in epidemiology (STROBE) statement: Guidelines for reporting observational studies. Int J Surg. 2014;12(12): 1495–1499. doi: 10.1016/j.ijsu.2014.07.013 25046131

[41] Noble CCA, Sturgeon JP, Bwakura-Dangarembizi M, Kelly P, Amadi B, Prendergast AJ. Postdischarge interventions for children hospitalized with severe acute malnutrition: a systematic review and meta-analysis. Am J Clin Nutr. 2021;113(3): 574–585. doi: 10.1093/ajcn/nqaa359 33517377 PMC7948836

[42] Aboud FE, Akhter S. A cluster-randomized evaluation of a responsive stimulation and feeding intervention in Bangladesh. Pediatrics. 2011;127(5): e1191–e1197. doi: 10.1542/peds.2010-2160 21502222

[43] Nahar B, Hossain I, Hamadani JD, Ahmed T, Grantham-McGregor S, Persson L. Effect of a food supplementation and psychosocial stimulation trial for severely malnourished children on the level of maternal depressive symptoms in Bangladesh. Child Care Health Dev. 2014;41(3): 483–493. doi: 10.1111/cch.12176 25040164

[44] Myatt M, Khara T, Schoenbuchner S, Pietzsch S, Dolan C, Lelijveld N, et al. Children who are both wasted and stunted are also underweight and have a high risk of death: A descriptive epidemiology of multiple anthropometric deficits using data from 51 countries. Arch Public Heal. 2018;76(1): 28. doi: 10.1186/s13690-018-0277-1 30026945 PMC6047117

[45] Wells JCK, Briend A, Boyd EM, Berkely JA, Hall A, Isanaka S, et al. Beyond wasted and stunted—a major shift to fight child undernutrition. Lancet Child Adolesc Heal. 2019;3(11): 831–834. doi: 10.1016/S2352-4642(19)30244-5 31521500

[46] Mwangome M, Ngari M, Fegan G, Mturi N, Shebe M, Bauni E, et al. Diagnostic criteria for severe acute malnutrition among infants aged under 6 mo. Am J Clin Nutr. 2017;105(6): 1415–1423. doi: 10.3945/ajcn.116.149815 28424189 PMC5445677

[47] Mwangome M, Ngari M, Bwahere P, Kabore P, McGrath M, Kerac M, et al. Anthropometry at birth and at age of routine vaccination to predict mortality in the first year of life: A birth cohort study in Bukina Faso. PLOS ONE. 2019;14(3): e0213523. doi: 10.1371/journal.pone.0213523 30921335 PMC6438502

[48] Stewart RC, Kauye F, Umar E, Vokhiwa M, Bunn J, Fitzgerald M, et al. Validation of a Chichewa version of the self-reporting questionnaire (SRQ) as a brief screening measure for maternal depressive disorder in Malawi, Africa. J Affect Disord. 2009;112(1–3): 126–34. doi: 10.1016/j.jad.2008.04.001 18504058

[49] Colman S, Stewart RC, Macarthur C, Kennedy N, Tomenson B, Creed F. Psychological distress in mothers of children admitted to a nutritional rehabilitation unit in Malawi—a comparison with other paediatric wards. Matern Child Nutr. 2015;11(4): 915–925. doi: 10.1111/mcn.12091 24224802 PMC6860349

[50] Stewart RC, Bunn J, Vokhiwa M, Umar E, Kauye F, Tomenson B, et al. A prospective study of psychological distress among mothers of children admitted to a nutritional rehabilitation unit in Malawi. Child Care, Heal Dev Heal Dev. 2011;37(1): 55–63. doi: 10.1111/j.1365-2214.2010.01111.x 20645996

