# Supplementary figures and images for: Pathways between caregiver body mass index, the home environment, child nutritional status, and development in children with severe acute malnutrition in Malawi
Source: PLoS One. 2021 Aug 23;16(8):e0255967. doi: 10.1371/journal.pone.0255967 (PMC8382172; doi:10.1371/journal.pone.0255967)

S1 Fig. Q–Q plots for variables in the structural equation model.

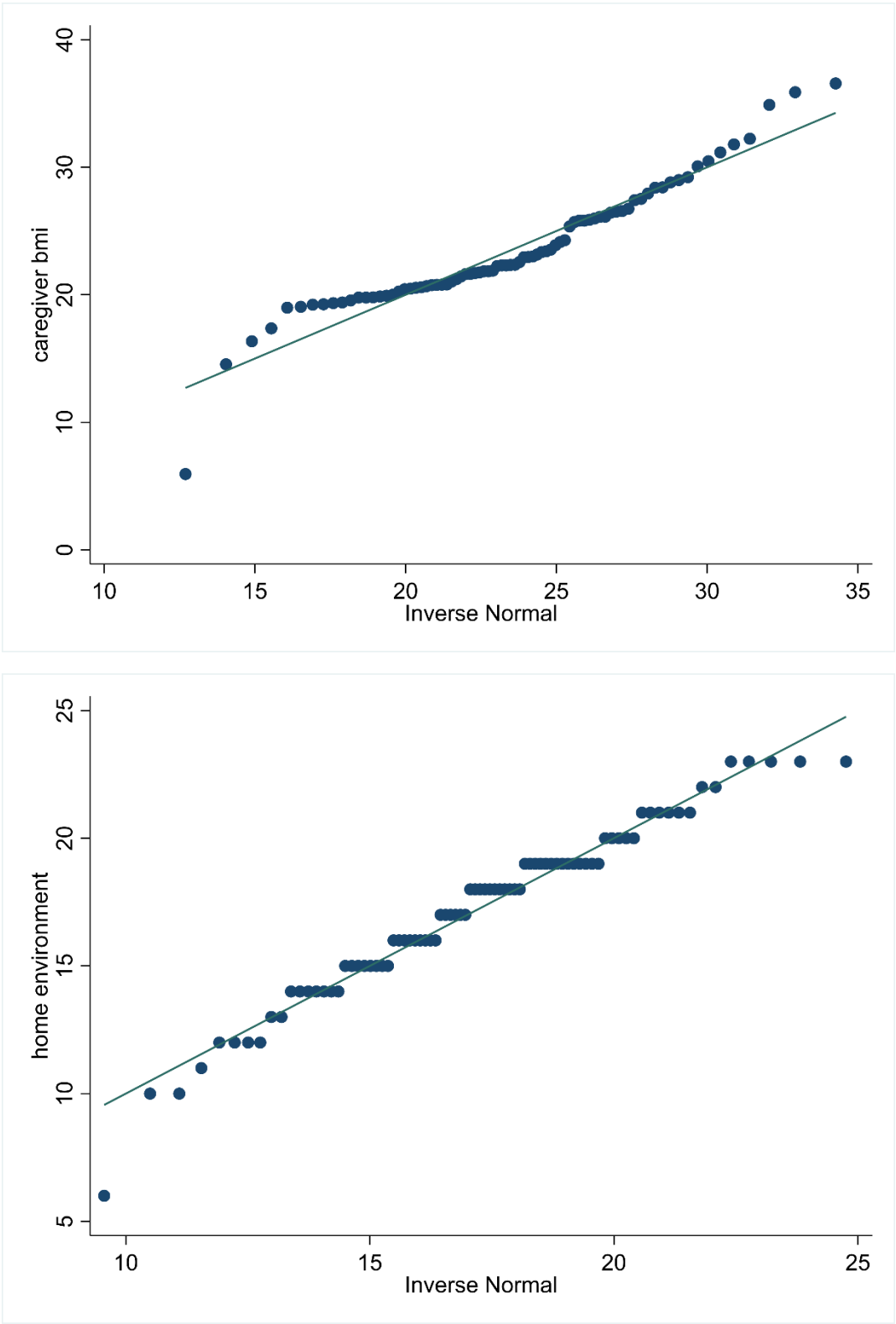

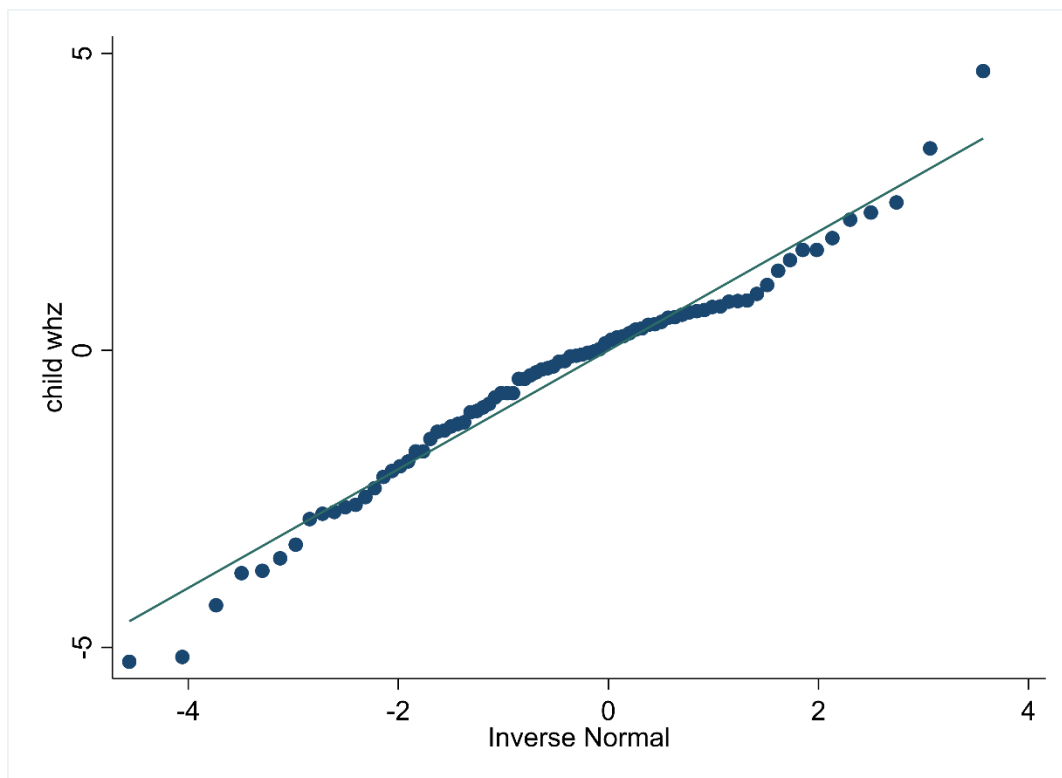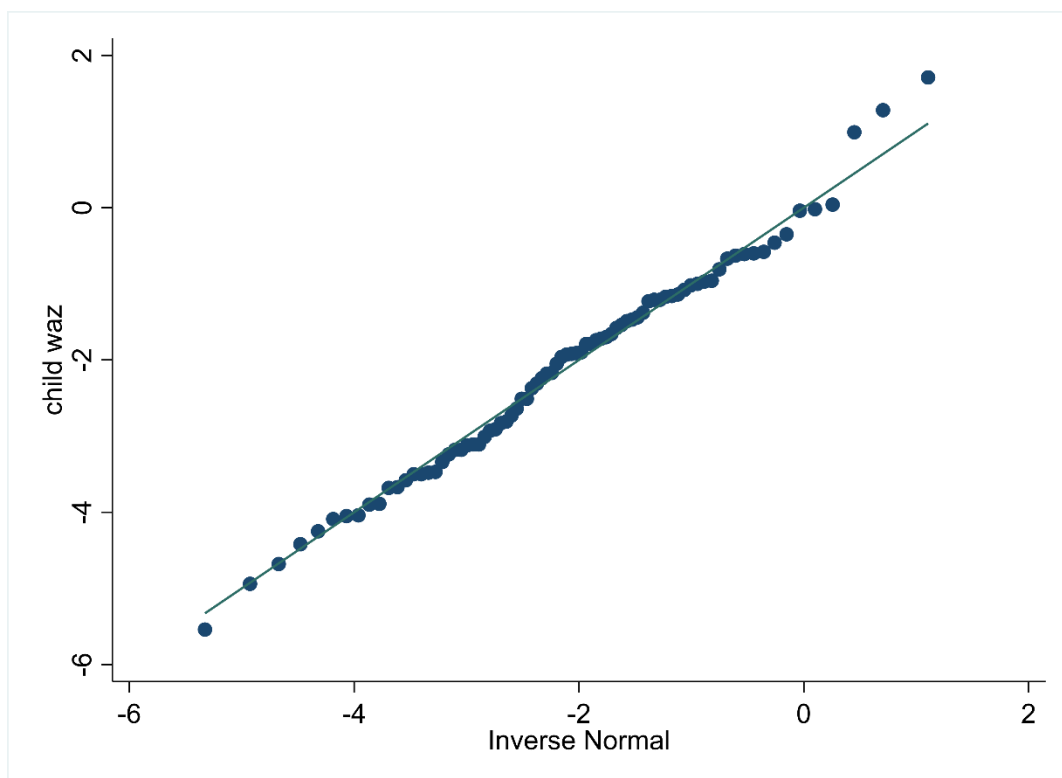

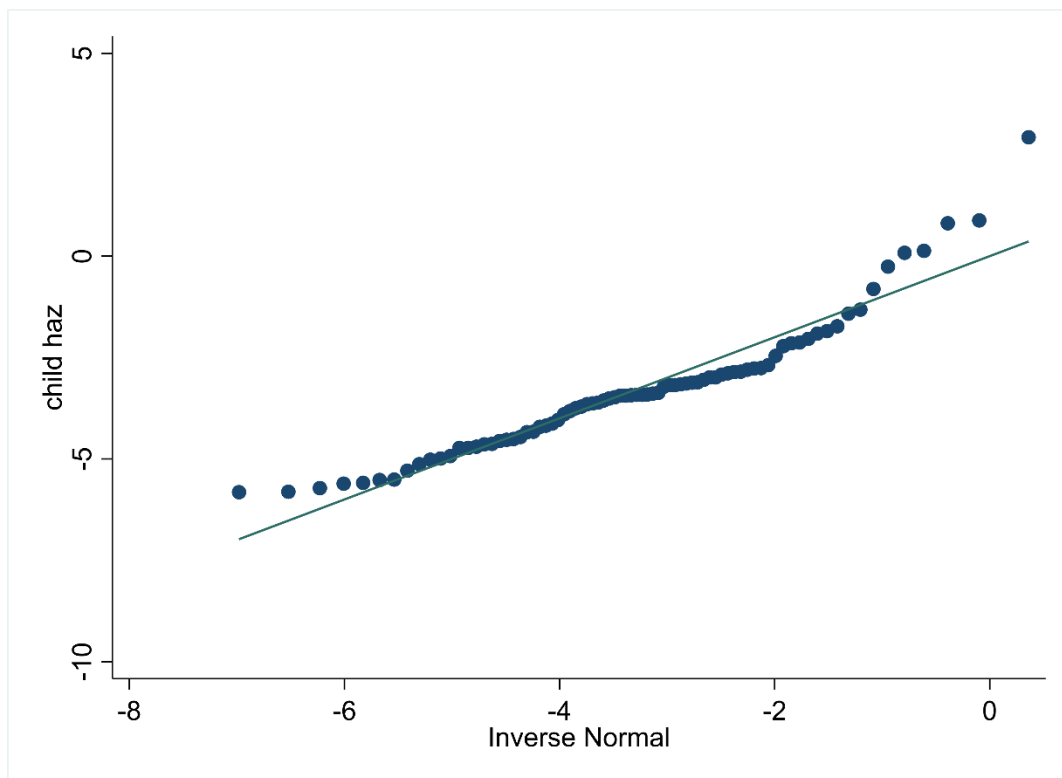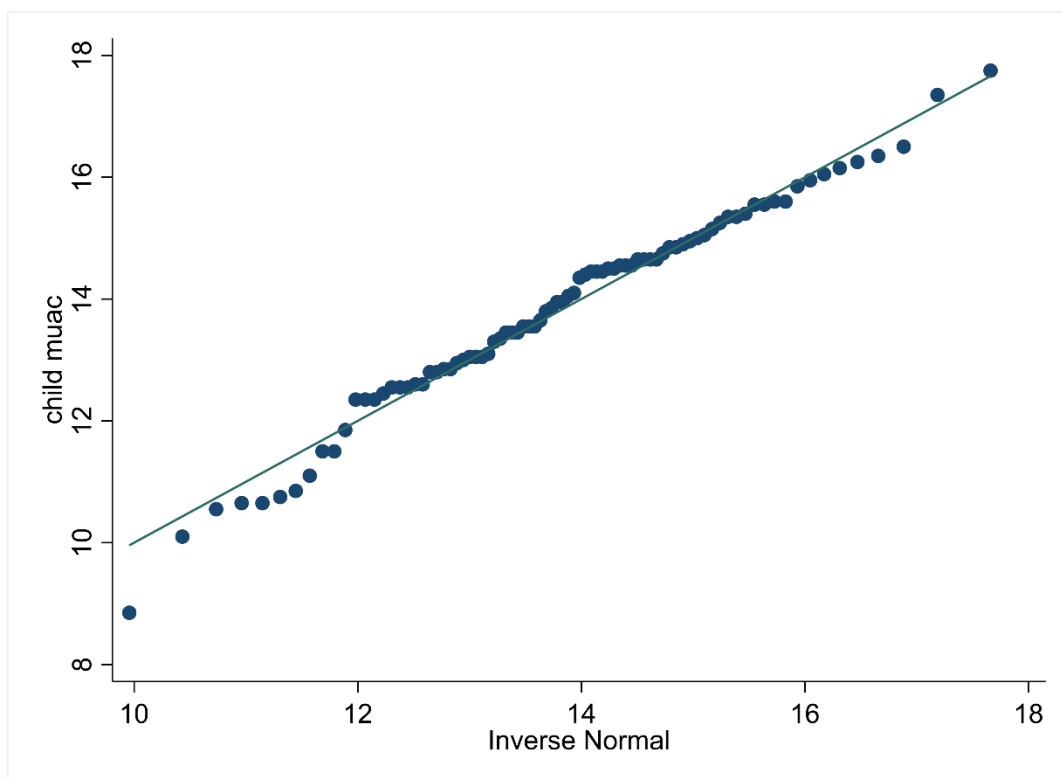

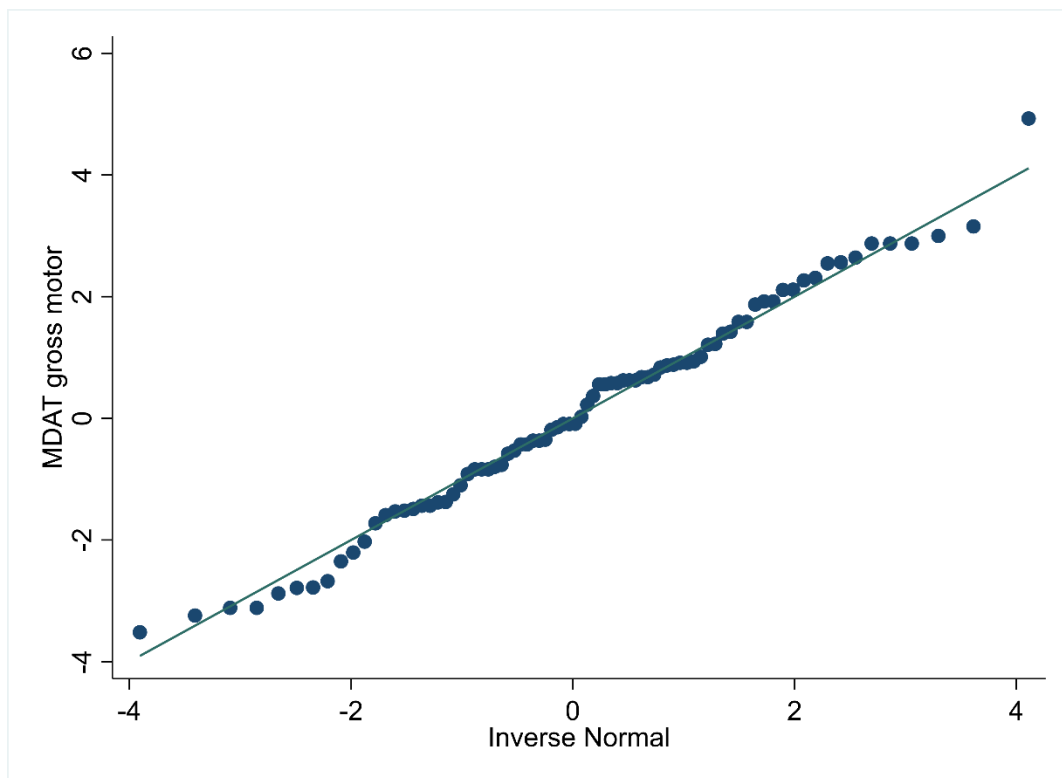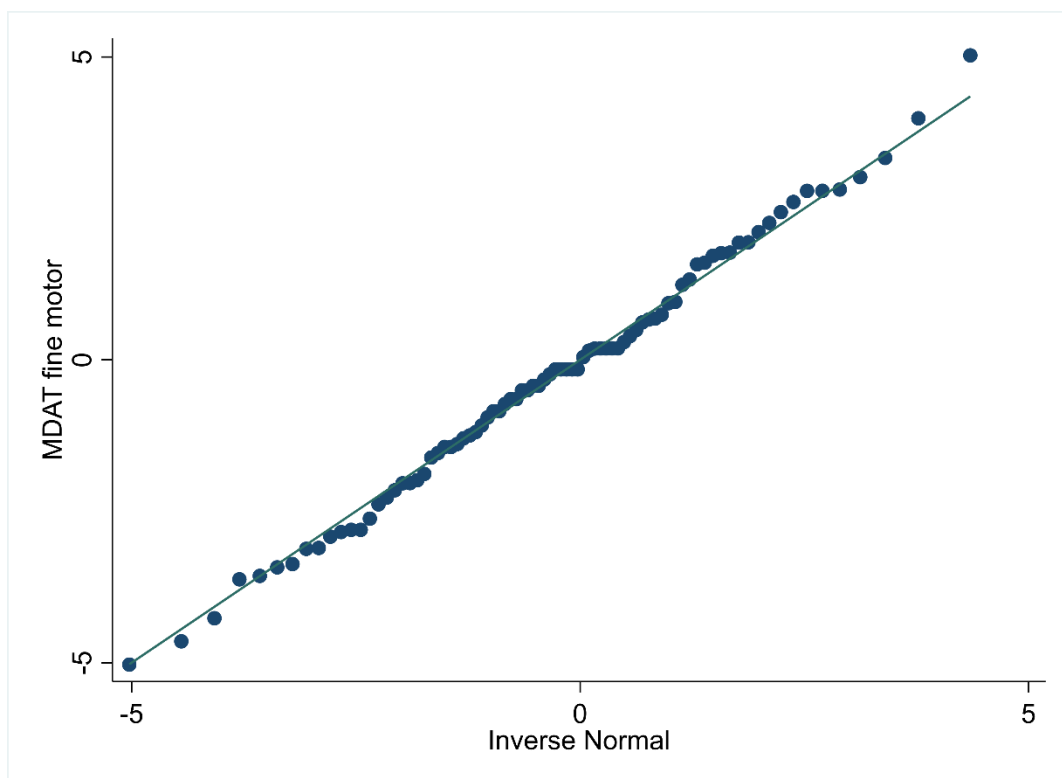

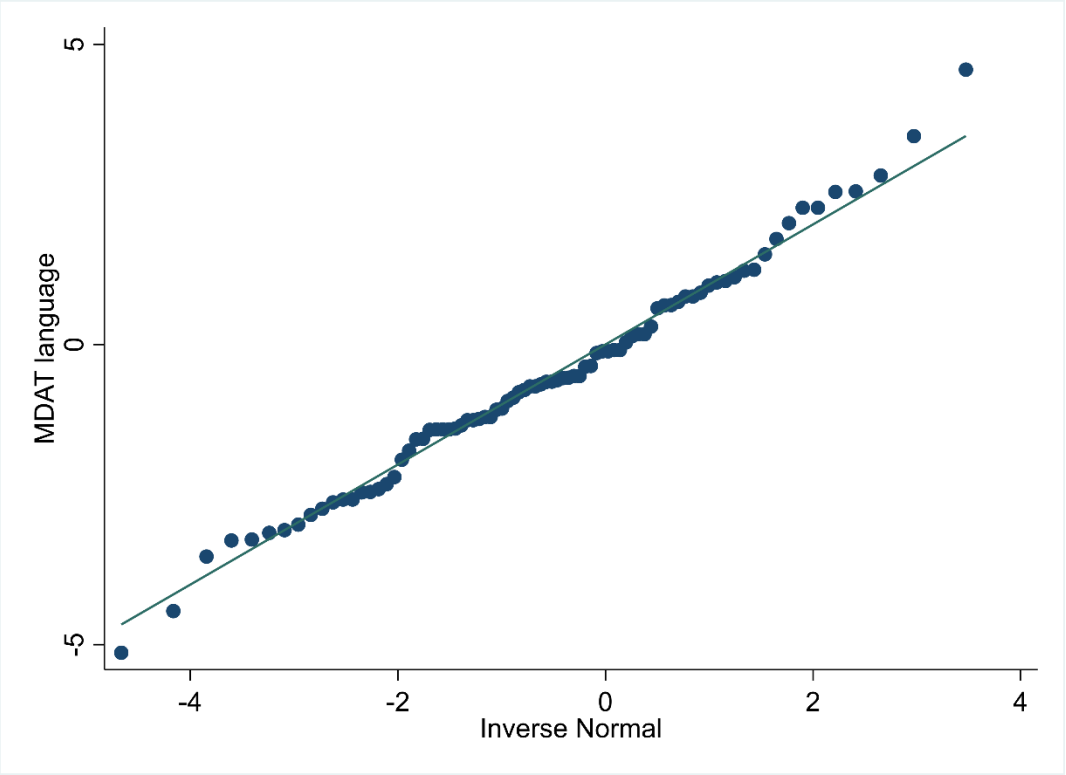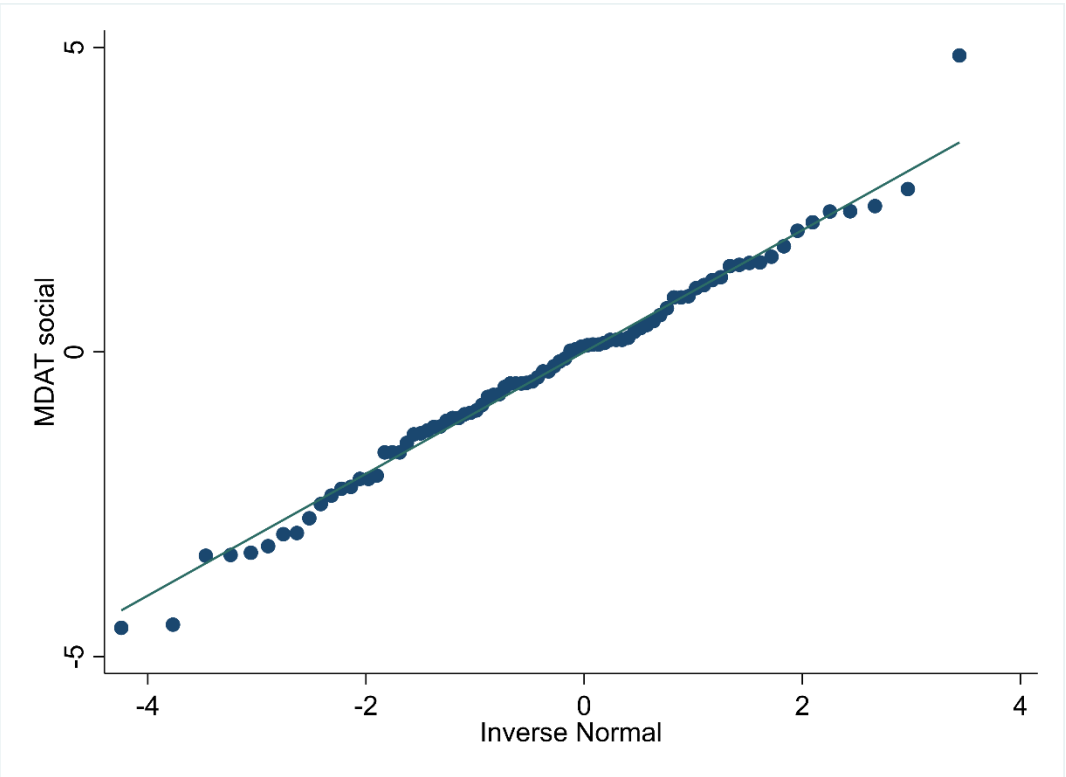

Supplement: S1 Fig — (PDF) [file pone.0255967.s002.pdf]

**S2 Fig. Study flow chart for structural equation model analysis.**

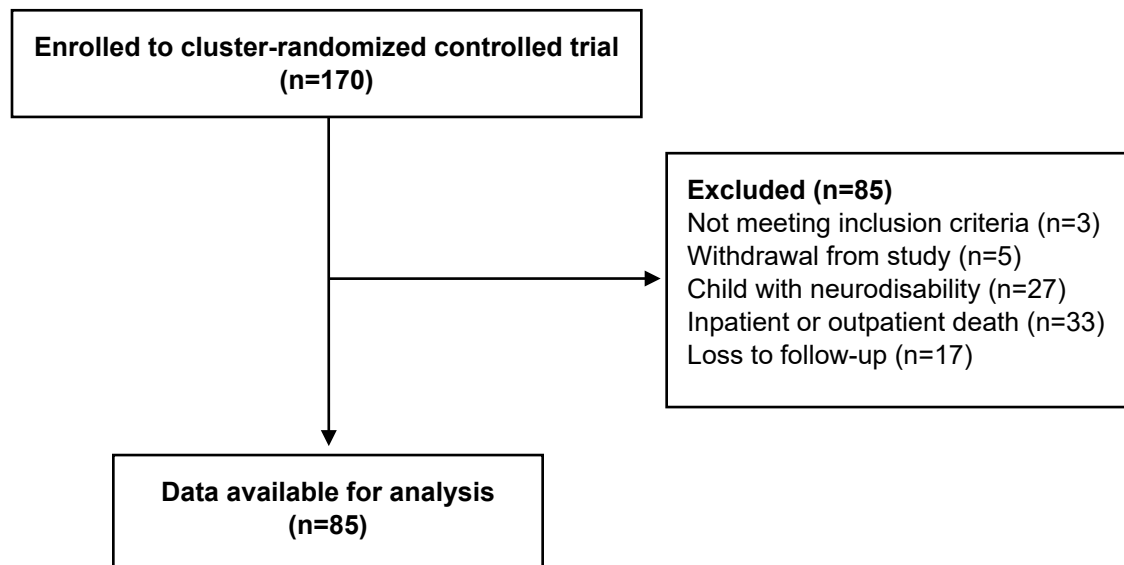

Supplement: S2 Fig — (PDF) [file pone.0255967.s003.pdf]
