# Supplementary material for: Pathways between caregiver body mass index, the home environment, child nutritional status, and development in children with severe acute malnutrition in Malawi
Source: PLoS One. 2021 Aug 23;16(8):e0255967. doi: 10.1371/journal.pone.0255967 (PMC8382172; doi:10.1371/journal.pone.0255967)
Supplement: S3 Fig — Caregiver body mass index (bmi); home environment according to the Home Observation for Measurement of the Environment Inventory; dietary diversity according to a 24-hour dietary recall; child nutritional status according to mid-upper arm circumference (muac); and development according to MDAT (Malawi Developmental Assessment Tool) z-scores. Estimates represent standardized beta-coefficients. Analysis adjusted for child HIV status, sex, and age. *p<0.05, **p<0.01. (PDF) [file pone.0255967.s004.pdf]

S3 Fig. Pathways between caregiver BMI, the home environment, child nutritional status according to MUAC, and development in children with SAM.

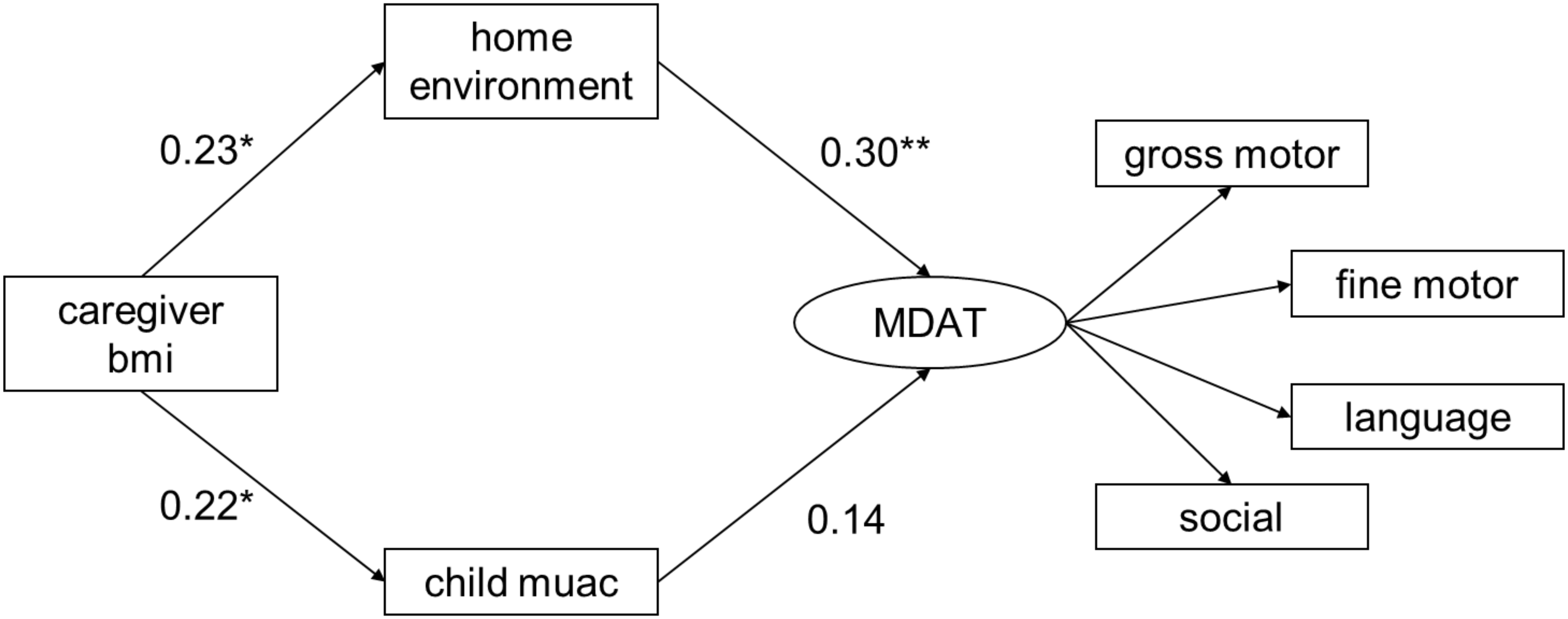

Caregiver body mass index (bmi); home environment according to the Home Observation for Measurement of the Environment Inventory; dietary diversity according to a 24-hour dietary recall; child nutritional status according to mid-upper arm circumference (muac); and development according to MDAT (Malawi Developmental Assessment Tool) z-scores. Estimates represent standardized beta-coefficients. Analysis adjusted for child HIV status, sex, and age. *p<0.05, **p<0.01.
