# Supplementary material for: Pathways between caregiver body mass index, the home environment, child nutritional status, and development in children with severe acute malnutrition in Malawi
Source: PLoS One. 2021 Aug 23;16(8):e0255967. doi: 10.1371/journal.pone.0255967 (PMC8382172; doi:10.1371/journal.pone.0255967)
Supplement: S1 Table — bmi, body mass index. haz, height-for-age z-score. home, Home Observation for Measurement of the Environment. MDAT, Malawi Developmental Assessment Tool. muac, mid-upper arm circumference. waz, weight-for-age z-score. whz, weight-for-height z-score. (PDF) [file pone.0255967.s005.pdf]

S1 Table. Missing data from participants in the structural equation model.

Variable	Missing (out of 85)	Percent missing
**caregiver bmi**	4	4.7
**home inventory score**	2	2.4
**child whz**	3	3.5
**child waz**	4	4.7
**child haz**	5	5.9
**child muac**	1	1.2
**MDAT**		
**gross motor**	3	3.5
**fine motor**	2	2.4
**language**	1	1.2
**social**	1	1.2

bmi, body mass index. haz, height-for-age z-score. home, Home Observation for Measurement of the Environment. MDAT, Malawi Developmental Assessment Tool. muac, mid-upper arm circumference. waz, weight-for-age z-score. whz, weight-for-height z-score.
