# Supplementary material for: Pathways between caregiver body mass index, the home environment, child nutritional status, and development in children with severe acute malnutrition in Malawi
Source: PLoS One. 2021 Aug 23;16(8):e0255967. doi: 10.1371/journal.pone.0255967 (PMC8382172; doi:10.1371/journal.pone.0255967)
Supplement: S2 Table — β, beta-coefficient (standardized). bmi, body mass index. MDAT, Malawi Developmental Assessment Tool. muac, mid-upper arm circumference. Analysis adjusted for child HIV status, sex, and age. (PDF) [file pone.0255967.s006.pdf]

S2 Table. **Standardized beta-coefficients and bootstrap results for direct pathways between caregiver BMI, the home environment, child nutritional status according to MUAC, and development in children with SAM.**

	**Structural equation modelling results**			**Bootstrap results**		
**Pathways**	**β**	**P-values**	**95% confidence interval**	**β**	**P-values**	**95% confidence interval**
**home environment**						
**caregiver bmi**	0.23	0.03	0.030, 0.44	0.23	0.01	0.050, 0.42
**child muac**						
**caregiver bmi**	0.22	0.04	0.010, 0.43	0.22	0.1	-0.040, 0.48
**MDAT**						
**home environment**	0.30	0.005	0.011, 0.43	0.30	0.006	0.084, 0.51
**child muac**	0.14	0.2	-0.071, 0.35	0.14	0.20	-0.073, 0.35

β, beta-coefficient (standardized). bmi, body mass index. MDAT, Malawi Developmental Assessment Tool. muac, mid-upper arm circumference. Analysis adjusted for child HIV status, sex, and age.
