# Supplementary material for: Pathways between caregiver body mass index, the home environment, child nutritional status, and development in children with severe acute malnutrition in Malawi
Source: PLoS One. 2021 Aug 23;16(8):e0255967. doi: 10.1371/journal.pone.0255967 (PMC8382172; doi:10.1371/journal.pone.0255967)
Supplement: S3 Table — bmi, body mass index. haz, height-for-age z-score. home, Home Observation for Measurement of the Environment. MDAT, Malawi Developmental Assessment Tool. muac, mid-upper arm circumference. waz, weight-for-age z-score. whz, weight-for-height z-score. (PDF) [file pone.0255967.s007.pdf]

S3 Table. Summary statistics of the variables in the hypothesized pathway model.

**Variable**	
**caregiver bmi (kg/m^2)**	23.5 ± 4.8
**home environment**	17.2 ± 3.4
**child whz**	-0.5 ± 1.8
**child haz**	-3.3 ± 1.6
**child waz**	-2.1 ± 1.4
**child muac**	13.8 ± 1.7
**MDAT**	
**gross motor**	0.1 ± 1.8
**fine motor**	-0.3 ± 2.1
**language**	-0.6 ± 1.8
**social**	-0.4 ± 1.7

bmi, body mass index. haz, height-for-age z-score. home, Home Observation for Measurement of the Environment. MDAT, Malawi Developmental Assessment Tool. muac, mid-upper arm circumference. waz, weight-for-age z-score. whz, weight-for-height z-score.
