# Supplementary material for: Pathways between caregiver body mass index, the home environment, child nutritional status, and development in children with severe acute malnutrition in Malawi
Source: PLoS One. 2021 Aug 23;16(8):e0255967. doi: 10.1371/journal.pone.0255967 (PMC8382172; doi:10.1371/journal.pone.0255967)
Supplement: S4 Table — β, beta-coefficient (standardized). haz, height-for-age z-score. mdat, Malawi Developmental Assessment Tool. muac, mid-upper arm circumference. waz, weight-for-age z-score. whz, weight-for-height z-score. (PDF) [file pone.0255967.s008.pdf]

S4 Table. Linear regression results for individual pathways in the hypothesized pathway model.

**Pathways**	**β**	**P-values**	**Adjusted R^2**
**home environment**			
**caregiver bmi**	0.24	0.037	0.043
**child whz**			
**caregiver bmi**	0.16	0.16	0.013
**child waz**			
**caregiver bmi**	0.28	0.015	0.064
**child haz**			
**caregiver bmi**	0.22	0.051	0.038
**child muac**			
**caregiver bmi**	0.22	0.048	0.037
**mdat gross motor**			
**home environment**	0.32	0.003	0.091
**child whz**	0.27	0.01	0.064
**child waz**	0.39	<0.001	0.14
**child haz**	0.26	0.02	0.055
**child muac**	0.25	0.02	0.052
**mdat fine motor**			
**home environment**	0.29	0.008	0.074
**child whz**	0.29	0.008	0.072
**child waz**	0.25	0.02	0.050
**child haz**	0.29	0.009	0.071
**child muac**	0.17	0.15	0.018
**mdat language**			
**home environment**	0.33	0.002	0.097
**child whz**	0.18	0.18	0.020
**child waz**	0.16	0.15	0.013
**child haz**	0.060	0.43	-0.0087
**child muac**	0.087	0.50	-0.0046
**mdat social**			
**home environment**	0.21	0.057	0.032
**child whz**	0.29	0.007	0.075
**child waz**	0.29	0.007	0.074
**child haz**	0.32	0.003	0.093
**child muac**	0.18	0.12	0.019

β, beta-coefficient (standardized). haz, height-for-age z-score. mdat, Malawi Developmental Assessment Tool. muac, mid-upper arm circumference. waz, weight-for-age z-score. whz, weight-for-height z-score.
